# Dynamics of Parasite Clearance in Cutaneous Leishmaniasis Patients Treated with Miltefosine

**DOI:** 10.1371/journal.pntd.0001436

**Published:** 2011-12-13

**Authors:** Thomas P. C. Dorlo, Pieter P. A. M. van Thiel, Gerard J. Schoone, Ymkje Stienstra, Michèle van Vugt, Jos H. Beijnen, Peter J. de Vries

**Affiliations:** 1 Division of Infectious Diseases, Academic Medical Center, University of Amsterdam, Amsterdam, The Netherlands; 2 Department of Pharmacy and Pharmacology, Slotervaart Hospital/The Netherlands Cancer Institute, Amsterdam, The Netherlands; 3 Parasitology Unit, KIT Biomedical Research, Royal Tropical Institute, Amsterdam, The Netherlands; Institut Pasteur de Tunis, Tunisia

## Abstract

Parasite loads were quantified in repeated skin biopsies from lesions of 2 patients with Old-World cutaneous leishmaniasis (CL) caused by *Leishmania major* and *L. infantum* during and after treatment with miltefosine. Miltefosine induced a rapid therapeutic effect on both infections with an initial decline of parasites of ∼1 log/week for the *L. major* infection. These observations illustrate the usability of quantifying parasite loads in skin lesions as a pharmacodynamic measure and quantitative descriptor of drug effect for CL supporting clinical assessment.

## Author Summary

The clinical evaluation of the ulcerated lesions in cutaneous leishmaniasis (CL) is both difficult and subjective. As a result, the evaluation of therapeutic efficacy of drugs for CL remains complicated. The relationship between dose and effect of antileishmanial drugs in CL is unclear and a good quantitative descriptor of drug effect has not yet been established. This report describes the use of quantifying the parasite load in repeated full-thickness skin biopsies from lesions of two patients with extensive Old-World CL (*Leishmania major* and *L. infantum*) who both were treated with miltefosine to demonstrate the dynamics of parasite clearance within CL lesions. Therapeutic effect of miltefosine was already noticeable directly after start of treatment, with a rapid, log-linear decline in parasite load in the skin biopsies of approximately 1 log/week for the *L. major* infection. These observations illustrate the applicability of quantifying parasite loads as a pharmacodynamic measure for CL supporting clinical assessment. The methodology described here might enable better evaluation and comparison of standard and new therapeutics in future randomized clinical trials for CL.

## Introduction

Miltefosine is an oral antileishmanial drug widely used in the management of visceral leishmaniasis (VL) in the Indian subcontinent [1]. There is increasing evidence on the efficacy of miltefosine in New-World cutaneous leishmaniasis (CL) [2]. In Old-World CL the applicability of miltefosine is documented in a few reports with *Leishmania major* as causative species [3]–[6]. The miltefosine dosage regimens used in CL are based on those established in Indian VL patients and lack a rational background [7].

Treatment can be given to speed up spontaneous healing of CL. Systemic treatment is indicated in ‘complex’ disease in patients with multiple lesions (>5), with lesions in cosmetically or functionally delicate areas, with lesions which are non-responsive to intralesional treatment or when mucocutaneous leishmaniasis may develop. Clinical assessment of the progress and healing of CL lesions remains difficult, certainly in complex CL cases, where definitive cure from a clinical perspective is determined up to 6 months post-treatment.

Nothing is known about the dose-effect relationship of miltefosine in CL, mainly because a good quantitative descriptor of drug effect has not yet been established. In an attempt to rationalize the treatment of CL and to support the clinical examination and follow-up, two patients with Old-World CL for whom systemic miltefosine treatment was indicated were followed-up by measuring the *Leishmania* parasite load in repeated skin biopsies during and after therapy.

## Materials and Methods

Two patients with cutaneous leishmaniasis described in this report both presented to the Unit of Tropical Medicine at the Academic Medical Center (AMC), Amsterdam, the Netherlands and were treated with miltefosine at the currently advised maximal total daily dose of 50 mg three times daily for a total of 28 days (Paladin Labs Inc, Montreal, Canada). Informed consent was obtained from both patients concerning the miltefosine treatment, the procedures described here and publication of the clinical descriptions and photographs.

In both patients, full thickness skin biopsies with the same diameter (2 mm) were taken repeatedly with a sterile disposable biopsy puncher from the active border of the same lesion at approximately the same location: at the edge of the inflammatory zone bordering the necrotic ulcer and always adjacent to the scar of the previous biopsy site. Biopsies were lysed using 950 µL L6-buffer, prior to extraction of RNA and DNA [8]. Parasite loads were measured by quantitative reverse-transcriptase real-time PCR (qRT-PCR) based on the detection of *Leishmania* 18S ribosomal RNA, which may allow for the detection of viable parasites [8]. *Leishmania* species were identified using the mini-exon repeat PCR method described by Marfurt et al. [9], with minor modifications [10]. Miltefosine plasma concentrations were measured by a validated liquid chromatography-coupled to tandem mass spectrometry method (LC-MS/MS) [11].

## Results

### Case Reports

#### Clinical description – Patient 1

In December 2008, a 53-year-old woman consulted the AMC, for a second opinion on treatment for persistent skin ulcers, elsewhere diagnosed as cutaneous leishmaniasis. Biopsy of one of the ulcers had shown Leishman-Donovan (LD) bodies, typed by PCR as L. major. Itraconazol (100 mg, twice daily) had no effect and was discontinued after 2.5 weeks.

**Figure 1:**
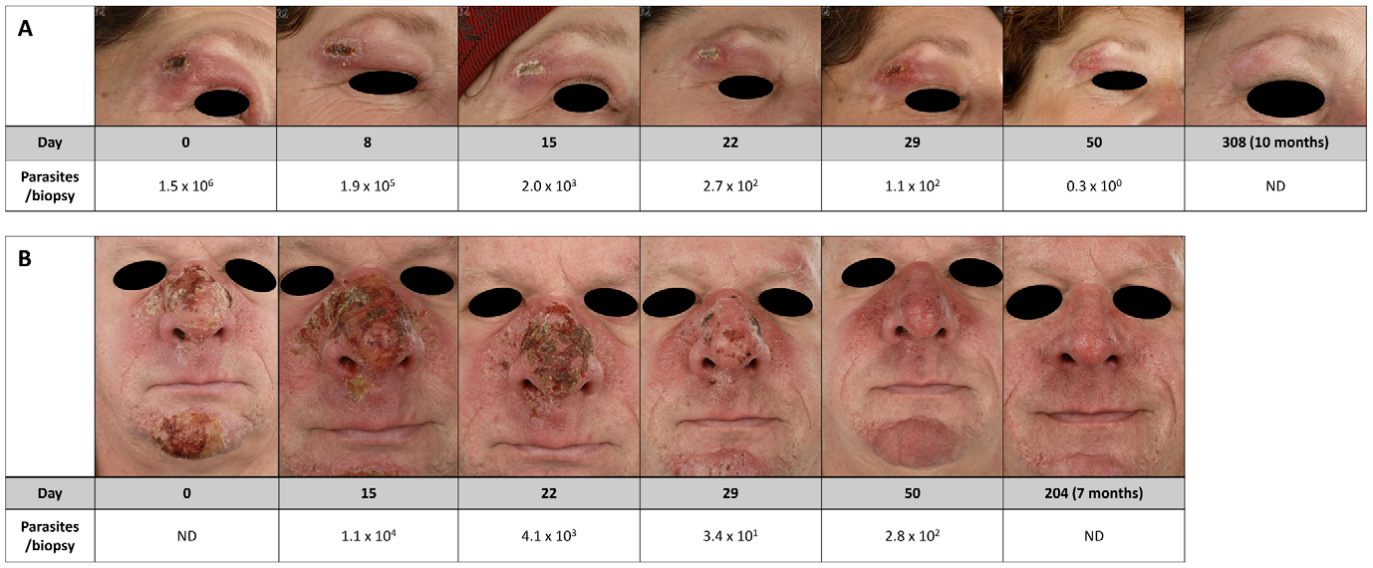
Clinical follow-up of CL lesions. Photographs of two selected lesions in relation to parasite loads in skin biopsies of two CL patients treated with miltefosine from the start of treatment until end of follow-up: A) *L. major* infection, B) *L. infantum* infection. ND = not determined.

In the past 5 years, she regularly made identical short visits to Morocco working as a travel guide. During the last visit, 2 months before presentation, papules appeared on trunk, face and upper extremities, which all progressed to ulcers. Until then she had been in good health apart from a period of treatment for presumed mixed connective tissue disease many years ago which was not further documented. On physical examination she was in a good condition with normal vital parameters and a body weight of 95 kg. A total of 15 ulcers (diameter 1–3 cm) were noted under the chin, on the eyelid, in the axillary fold, on upper and lower arms and on the trunk. No lymphadenopathy or other abnormalities were present. The purulent ulcer on the eyelid was surrounded by a red edematous swelling and covered by a crust. The high number of disseminated lesions and the facial involvement required systemic treatment. She was advised to take oral miltefosine (50 mg, thrice daily for a total of 28 days), since standard parenteral antimony treatment is relatively toxic and cumbersome. Because of secondary bacterial infection of some of the ulcers with *Staphylococcus aureus*, oral azithromycine was prescribed (first day 1500 mg, followed by 6 days 500 mg daily). On the first day of treatment, she experienced diarrhea, and thereafter only stomach aches when the miltefosine capsules were not taken concurrently with food. At the end of treatment, all lesions were reduced in size and started to re-epithelialize. Seven weeks after the end of treatment, all lesions had evolved into scars with complete re-epitheliazation. At 6 months, no recurrence was seen and no parasites could be detected in a biopsy from a former lesion by qRT-PCR (Figure 1A).

#### Clinical description - Patient 2

In June 2008, a 54-year-old man known with acne, took a 3-week holiday at the Canary Island of Tenerife and in Northern Spain, during which he experienced a painful open sunburn on the nose. Two months later, non-healing papules and exudative crustated erosions appeared on the nose and chin, which increased in size and quantity and were surrounded by an erythematous swelling. He was diagnosed as having rosacae with secondary bacterial infection and treated with metronidazole and fucidin cream. A biopsy was taken from the intranasal tissue, because of obstruction of the left nasal passage. This revealed a non-specific edematous inflammatory and fibrinous reaction. In combination with the then present arthralgia of the right knee, Wegener's disease was suspected but was consecutively excluded on the basis of serology, as well as other autoimmune disorders. In January 2009, infiltrated erythematous swellings had expanded to the upper lip and cheek, as also small erythematous erosions appeared above the eyebrow and on the scalp. A biopsy of the chin lesion showed LD bodies. He was subsequently referred to the AMC for further management. The skin lesions were still as described above, while the mucosa of the left side of the nasal septum showed boggy indurations with purulent secretion. His body weight was 109 kg (height 180 cm). Hematology and liver function tests were normal. PCR typing of skin biopsies from the lesions on nose and chin demonstrated a *L. infantum* infection. Because of extensive skin involvement, he was treated systemically with oral miltefosine (50 mg, thrice daily for a total of 28 days).

During treatment he experienced many episodes of diarrhea and vomiting, up to four times a day, but these complaints subsided when miltefosine was taken together with food. Although the lesions seemed to ameliorate quickly after the start of treatment, definite cure could only be concluded on follow-up at 7 months after initiation of therapy (Figure 1B).

### Laboratory Investigations

**Figure 2:**
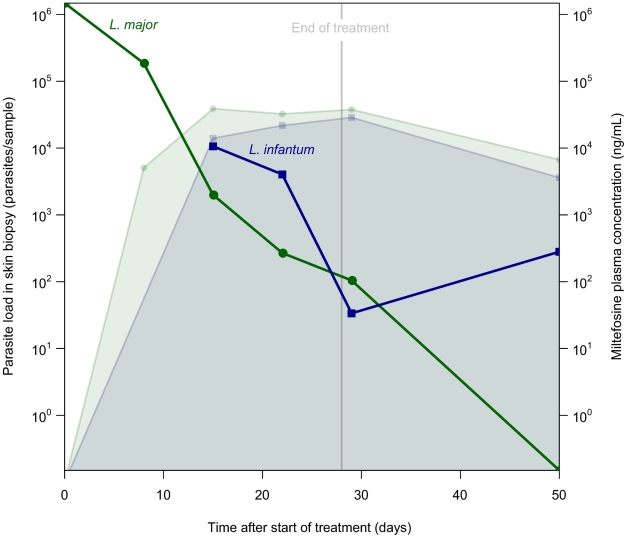
*Leishmania* parasite loads in skin biopsies from lesions of two CL patients treated with oral miltefosine. Miltefosine was given (50 mg thrice daily) for a total of 28 days. Miltefosine trough plasma concentrations (ng/mL) are indicated by the colored filled areas. The Leishmania qRT-PCR values of the skin biopsies (parasites/sample) are indicated by colored lines and dots. The green line, circles and filled area represent Patient 1 (*L. major*), while the blue line, squares and filled area represent Patient 2 (*L. infantum*).

The time courses of *Leishmania* parasite loads in the skin biopsies quantified with qRT-PCR are shown in Figure 2. The initial parasite load for *L. major* at start of treatment was 1.5×10^6^, while the first recorded parasite load for *L. infantum* at day 15 of treatment was 1.1×10^4^. At the end of treatment (day 28), skin biopsies taken from both patients still revealed the presence of parasite RNA/DNA as measured by qRT-PCR: 104 and 34 parasites per skin biopsy in the lesions of the *L. major* and *L. infantum* infection, respectively. The lesion of Patient 1 (*L. major*) was parasite-free after 50 days after start of treatment with a rapid parasite clearance rate of ∼1 log/week and remained parasite-free during follow-up examinations. The initial parasite clearance rate for Patient 2 (*L. infantum*) between day 15 and 22 of treatment appeared to be lower (<0.5 log/week) compared to the *L. major* infection, although on average in the last two weeks of treatment (day 15–29) a similar clearance rate of ∼1 log/week was observed in the two infections. Patient 2, however, had a slight upsurge of parasites on day 50 after start of treatment, despite clinical improvement of the lesions. Unfortunately, no skin biopsy of Patient 2 was available from the follow-up period. Miltefosine kept accumulating until the end of treatment and plasma trough concentrations had increased on day 28 (last day of treatment) to 38 µg/mL and 29 µg/mL for Patient 1 and Patient 2, respectively (Figure 2).

## Discussion

This report describes two patients with extensive Old-World CL: one with *L. major* infection which suggested dissemination or at least multiple inoculations and the other with *L. infantum* infection of facial skin with collateral swelling of the nasal mucosa. Both patients were treated with miltefosine and both showed a parallel log-linear decline of the parasite load in skin biopsies.

The described cases of Old-World CL originated from Northern Africa and the Mediterranean Basin caused by *L. major* and *L. infantum*, respectively. *L. infantum* has been described as a sporadic cause of CL [12]–[14]. To our knowledge, this case is the first reported *L. infantum*-related CL primarily treated with miltefosine.

The initial parasite load in the skin lesions was high but probably in the same order of magnitude for both patients (parasite load on day 0 was missing for the *L. infantum* infected patient). The repeated skin biopsies showed a rapid log-linear decline after start of miltefosine treatment, comparable to the decline rate of parasites in the blood of a previously miltefosine-treated VL patient [15]. The reproducible nature of the results was not completely expected, since we had anticipated a greater variability of parasite density among the repeated biopsies. This suggests that the parasite density is rather homogenously spread in the inflammatory zone that surrounds the necrotic ulcer and that the depth of the biopsy does not require calibration, as long as the diameter of the punch hole is calibrated and includes the full-thickness skin, since parasites accumulate in the upper layer of the dermis.

Therapeutic effect of miltefosine was thus already noticeable directly after start of treatment, although miltefosine-levels had not reached steady-state yet. Surprisingly, at the end of treatment (day 28), the examined skin biopsies still revealed the presence of *Leishmania* parasites in the lesions of both patients, indicating that complete elimination of the parasites in the lesion does not occur within the period of drug administration. After an initial decrease of the parasite load of *L. infantum*, this slightly increased again on day 50, possibly indicating a slower parasite clearance rate in the lesion of this patient. This may also have been caused by variability in parasite densities among the biopsy sites. Unfortunately, no further biopsies were taken to confirm complete parasite clearance from the lesion. Old-World CL is a slow healing condition of which clinical evaluation can pose difficulties in the first 3 months and is also known to be self-healing. The purpose of treatment of CL is to speed up spontaneous cure. Here we illustrate that parasite clearance rates from skin lesions, which can be assessed with qRT-PCR, can be used as a pharmacodynamic measure or ‘biomarker’ in future randomized clinical trials on CL.

Miltefosine has an extremely long terminal half-life of over 30 days, resulting in high plasma concentrations of miltefosine for a prolonged period of time after ending the 28-day therapy [7]. Whether the continued parasite clearance after discontinuation of drug administration is due to long-time residence of miltefosine concentrations after end of therapy, due to induced immunological response prompted by the therapy or rather natural evolution, is unknown. It is important to note that CL can be caused by a wide variety of *Leishmania* species and a high variability in sensitivity to miltefosine has previously been shown *in vitro* between these different species [16], however various host-related factors make it difficult to extrapolate these observations to the *in vivo* sensitivity of the parasite. The most commonly seen side-effects of miltefosine, mild to moderate vomiting and diarrhea, are probably related to drug-intake and not to drug plasma concentrations. Concurrent intake of a fatty meal largely reduced gastro-intestinal complaints in both patients.

In conclusion, a rapid therapeutic effect was observed in two patients with respectively *L. major*- and *L. infantum*-related CL after initiation of miltefosine treatment. Repeated quantifications of the parasite load in skin biopsies showed a rapid, log-linear, decline of approximately 1 log/week in the *L. major*-related CL and lesions were free of parasites 50 days after start of miltefosine treatment. The *L. infantum*-related CL seemed to respond more slowly, possibly due to mucosal involvement, but definite cure could eventually be determined at 7 months follow-up. To establish the role of miltefosine in the treatment of Old-World CL, more and better designed randomized controlled clinical trials employing pharmacokinetics and pharmacodynamics are needed.
